# Rotavirus RNA chaperone mediates global transcriptome-wide increase in RNA backbone flexibility

**DOI:** 10.1093/nar/gkac738

**Published:** 2022-09-05

**Authors:** Aaztli Coria, Anastacia Wienecke, Michael L Knight, Daniel Desirò, Alain Laederach, Alexander Borodavka

**Affiliations:** Department of Biochemistry and Biophysics, University of North Carolina, Chapel Hill, NC 27599, USA; Department of Biology, University of North Carolina at Chapel Hill, Chapel Hill, NC 27599, USA; Department of Biology, University of North Carolina at Chapel Hill, Chapel Hill, NC 27599, USA; Curriculum in Bioinformatics and Computational Biology, University of North Carolina at Chapel Hill, Chapel Hill, NC 27599, USA; Department of Biochemistry, University of Cambridge, Cambridge, UK; Department of Biochemistry, University of Cambridge, Cambridge, UK; Department of Biology, University of North Carolina at Chapel Hill, Chapel Hill, NC 27599, USA; Curriculum in Bioinformatics and Computational Biology, University of North Carolina at Chapel Hill, Chapel Hill, NC 27599, USA; Department of Biochemistry, University of Cambridge, Cambridge, UK

## Abstract

Due to genome segmentation, rotaviruses must co-package eleven distinct genomic RNAs. The packaging is mediated by virus-encoded RNA chaperones, such as the rotavirus NSP2 protein. While the activities of distinct RNA chaperones are well studied on smaller RNAs, little is known about their global effect on the entire viral transcriptome. Here, we used Selective 2′-hydroxyl Acylation Analyzed by Primer Extension and Mutational Profiling (SHAPE-MaP) to examine the secondary structure of the rotavirus transcriptome in the presence of increasing amounts of NSP2. SHAPE-MaP data reveals that despite the well-documented helix-unwinding activity of NSP2 *in vitro*, its incubation with cognate rotavirus transcripts does not induce a significant change in the SHAPE reactivities. However, a quantitative analysis of mutation rates measured by mutational profiling reveals a global 5-fold rate increase in the presence of NSP2. We demonstrate that the normalization procedure used in deriving SHAPE reactivities from mutation rates can mask an important global effect of an RNA chaperone. Analysis of the mutation rates reveals a larger effect on stems rather than loops. Together, these data provide the first experimentally derived secondary structure model of the rotavirus transcriptome and reveal that NSP2 acts by globally increasing RNA backbone flexibility in a concentration-dependent manner.

## INTRODUCTION

Virions of rotaviruses (RV), a large group of important human and animal pathogens, contain 11 distinct dsRNA molecules, or genomic segments, all of which are essential for rotavirus infectivity (1–3). Each dsRNA segment serves as a template for transcribing differently sized (0.67–3.4 kb) protein-coding + ssRNA transcripts (mRNAs) that can fold into regulatory structures, such as those predicted to be found in the 5′ and 3′ untranslated regions (UTRs) (4,5). In addition, consensus sequences found in 5′ and 3′ terminal regions of these segments also have important regulatory roles in ensuring proper viral infection, such as recruiting viral proteins to enhance viral translation (6,7). Li *et al.* provided one of the most comprehensive studies of rotavirus RNAs based on the minimum free energy consensus structures deduced from multiple sequence alignments and covariation analysis of group A rotavirus (RVA) strains (5). This study revealed multiple conserved RNA regions present in all RNA segments that were proposed to fold into regulatory elements. However, in the Li *et al* study, structure probing validation of generated models was only carried out for the smallest segment 11 transcript. While purified infectious rotavirus particles contain eleven dsRNA molecules thus precluding its chemical structure probing *in virio*, structural analysis of a complete rotavirus transcriptome with single-nucleotide precision would enable identification of RNA structures for further functional validation through recently established reverse genetics studies (8). Structural characterization of multiple RNA viruses have provided insights into previously unknown functional regulatory motifs (9,10).

Rotaviruses encode an RNA chaperone, NSP2, that is believed to be involved in RNA selection and stoichiometric segmented genome packaging (3,11). Single-molecule fluorescence studies of NSP2-mediated RNA remodeling with short (<50 nts) RNA substrates indicate that NSP2 binds single-stranded RNA with nanomolar affinity, causing transient helix destabilization and melting of its secondary structure (12,13). These studies also revealed that NSP2 octamers can bind to RNA stem-loops in both open and closed conformations, raising further questions about the mode of action of NSP2 as an RNA chaperone that promotes inter-segment RNA interactions in RVs (13). While these studies were important for our understanding of the RNA chaperone-mediated RNA annealing, there is no direct evidence of structural reorganization of the RVA RNA upon NSP2 binding. NSP2 binding itself poses a major challenge for modelling RV RNA structures as it is not known how multivalent RNA chaperones alter RNA folding on a transcriptome level (14). The impact of NSP2’s helix-destabilizing activity has yet to be investigated using RV RNA at the transcriptome level.

RNA folding is mediated by base-pairing that forms secondary structures, which play a myriad of fundamental regulatory roles, including in gene expression (9,16,17). To experimentally interrogate RNA structure, chemical structure probing coupled with thermodynamic modeling provides single nucleotide resolution information with transcriptome scale throughput (18,19). This is particularly pertinent for viruses, whose RNA genomes, or ssRNA precursors of dsRNA genomes, can be directly translated into viral proteins, or participate in the assembly of a nascent virus (20,21). Thus, experimentally investigating RNA structure with chemical probing allows us to identify potentially new and important roles for RNA structure in rotaviruses.

Here, we provide experimentally validated structure models of a complete rotavirus RNA pre-genome probed by selective 2′-hydroxyl acylation analyzed by primer extension (SHAPE) and mutational profiling (MaP) analysis (19,22). We identified multiple conserved structured regions with low SHAPE reactivity located in the 5′ terminal regions of RV RNAs. We show that the RNA chaperone NSP2 binding to RV transcripts dramatically increases mutational rates induced by the SHAPE reagent, but has no effect on the computed SHAPE reactivity, suggesting a global effect on backbone flexibility with minimal structural rearrangement. Analysis of SHAPE-informed secondary structure models in the presence of NSP2 reveals that despite its promiscuous RNA binding mode, its binding preferentially affects base-paired elements, consistent with the RNA chaperone activity. These findings support a model in which promiscuous binding of NSP2 to the rotavirus transcriptome allows this chaperone to uniformly increase the flexibility of the RNA backbone, thus leading to the increased accessibility of RNA towards the establishment of inter-molecular contacts.

## MATERIALS AND METHODS

### Rotavirus A transcripts

pUC19 clones encoding for segments 1–11 of bovine rotavirus A (strain RF, G6P6[1]) (13,23) were used for *in vitro* transcription reactions. Sequences for the 11 segments are listed in Supplementary data file 1 in SNRNASM format. The plasmids coding for segments 2 and 8 were digested using BbsI, the plasmid coding for segment 10 was linearized with BsaI, and the plasmids coding for the remaining segments were linearized using BsmBI restriction enzymes, following the manufacturer's protocol (NEB). Following linearization, the digestion product was purified via gel extraction (QiaQuick Gel Extraction Kit). Each RV segment was then transcribed using 1 μg of linearized DNA with HiScribe T7 from NEB at 37°C for 3 h. The transcription reaction was subsequently treated with Turbo DNAse (ThermoFisher) for 30 min at 37°C. The RNA was purified using Purelink RNA columns (ThermoFisher) and eluted in 30 μl of nuclease free water. The quality and size of each transcript were assessed on a 1.2% denaturing MOPS formaldehyde agarose gel. Prior to loading on the gel RNA was incubated at 65°C (10 min) in formamide-containing denaturing RNA loading buffer (ThermoFisher).

### NSP2 expression and purification

A sequence-verified pET-28b-NSP2 construct (13) was used for protein expression, as described in (24). Ni-affinity-purified NSP2 fractions were further purified over a HiTrap SP cation-exchange column (Cytiva) and the concentrated peak fractions were resolved on a Superdex 200 10 × 300 GL column. The column was pre-equilibrated with RNAse-free SEC buffer (25 mM HEPES-Na, pH 7.5, 150 mM NaCl) to ensure high purity and homogeneity of the preparation. The peak fraction was determined to be at least 99% pure by SDS-PAGE, with a characteristic 260 nm/280 nm absorbance ratio of <0.57, suggesting that the purified protein was pure from contaminating nucleic acids.

### Structure probing using selective 2′-hydroxyl acylation analyzed by primer extension (SHAPE) and mutational profiling (SHAPE-MaP)

For single RNA experiments the single RNA segments were diluted to a final concentration of 260 nM and incubated at 65°C for 5 min then slowly cooled back to room temperature for 30 min before then being kept at 4°C. For transcriptome wide experiments each RV segment was diluted to a concentration of 25 nM, incubated at 65°C for 5 min then slowly cooled back to room temperature. The segments were then pooled, resulting in an equimolar mix of all 11 RV segments. Stock NSP2 was diluted using 5 mM HEPES at pH 7 to concentrations required for the titrations described; 40 μM, 20 μM and 10 μM. The RNA was then buffered in the following conditions: 1 mM MgCl_2_, 200 mM NaCl, and 200 mM HEPES (pH 7). The diluted NSP2 and RNA were mixed in a 1:1 volume. For the no protein condition, 5 mM HEPES was added at equal volume to the RNA. The RNA:NSP2 samples were then incubated at 37°C for 30 min prior to probing. For probing, RNA:protein mix were incubated with 10% volume of 125 mM 5-nitroisatoic-anhydride (5NIA), dissolved in dimethyl sulfoxide (DMSO), to give a final concentration of 12.5 mM 5NIA. For 1M7 (1-methyl-7-nitroisatoic anhydride) treated samples, RNA was treated with a 10% volume of 100 mM 1M7 for a final concentration of 10 mM 1M7. Untreated samples were incubated with 10% DMSO. Both modified and untreated samples were vortexed and then incubated at 37°C for 5 min. RNA was subsequently purified using the Qiagen MinElute columns and eluted in 30 μl of nuclease-free water.

### Dimethyl sulfate (DMS) probing of transcripts for segment-specific amplification

Rotavirus segment 11 RNA was refolded as described above. The RNA was diluted to a final concentration of 250 nM in the RNA folding buffer (5 mM HEPES-Na pH 7.5, 0.5 mM MgCl_2_, and 100 mM NaCl). The diluted RNA was then mixed with an equal volume of either 20 mM NSP2 and samples were incubated at 37°C for 30 min. For DMS probing, 1 M bicine pH 8.0 was added to each sample give a final concentration of 200 mM. Samples were incubated at 37°C for 1 min with 10% DMS in ethanol to give a final DMS concentration of either 0.5% or 0.1% (v/v). Control samples were incubated with an equivalent volume of ethanol. Reactions were quenched by addition of 1 M DTT to a final concentration of 167 mM. RNA was purified using the Zymo RNA Clean and Concentrator 5 kit. Samples were then prepared using our segment specific library preparation protocol as described below.

### In-cell structure probing using SHAPE-MaP

MA104 cells were grown in T25 flasks in DMEM-high glucose (Sigma), supplemented with 2 mM GlutaMAX (Gibco), 100 U/ml of Penicillin-Streptomycin (Gibco), and 10% fetal calf serum (FCS) until confluent. Cells were washed by incubating with FCS free media at 37°C for 30 min. Group A rotavirus (RVA), strain RF (bovine) was activated by incubating with 1.5 μg/ml of trypsin for 30 min at 37°C, as previously described (25). Infections were performed in 3 ml of FCS-free media for 2 h, at 37°C, at an MOI of 1.4. Following the initial incubation, an additional 7.5 ml of FCS-free media was added and cells were incubated for a further 6 h at 37°C. Cells were washed twice with PBS and detached by incubation with trypsin. Detached cells were pelleted at 500 × g for 5 min and resuspended in 1.8 ml of 200 mM HEPES-Na pH 7.5 in PBS. The sample was then split in two and incubated with a 10% (v/v) of 250 mM 5NIA (25 mM final concentration) or DMSO at 37°C for 10 min. The reaction was quenched by the addition of 1 M DTT to a final concentration of 125 mM, and RNA was purified using the Monarch Plasmid Miniprep Kit (NEB).

### Tagmentation-based library preparation and next generation sequencing

First stand cDNA synthesis of treated RNA was carried out as previously described (22). Briefly, 29 μl of purified RNA was added to 100 ng Random Primer 9 (NEB), and 0.2 mM of each dNTP. This mix was then incubated at 65°C for 5 min before being transferred to ice, followed by addition of 50 mM Tris (pH 8.0), 75 mM KCl, 10 mM DTT, and 6 mM MnCl_2_ and 2 μl of SuperScript II Reverse Transcriptase (Invitrogen), supplemented with 20 U of murine RNase inhibitor (NEB) to achieve a final volume of 40 μl. The reaction was incubated at 25°C for 10 min, then 42°C for 3 h, then 70°C for 15 min. First strand cDNA was desalted using G50 columns (GE). The desalted product was then introduced into a second strand synthesis reaction. The cDNA second strand was generated using mRNA Non-Directional Second Strand Synthesis module (NEB). Double stranded DNA was then cleaned up using Ampure XP purification beads and eluted in 30 ul of water and quantified using a Qubit dsDNA HS Assay (Invitrogen). Double-stranded DNA was fragmented, repaired, and adaptor ligated for sequencing library preparation using the NEBNext^®^ Ultra™ II DNA Library Prep Kit for Illumina^®^. Library quality was assessed using the High Sensitivity Bioanalyzer protocol (Agilent). Libraries were then loaded on an Illumina MiSeq at 10 pM and run at 300 cycles (150 × 2) or 600 cycles (300 × 2).

### Segment-specific library preparation and next generation sequencing

500 ng of total RNA was combined with 2 pmol of reverse transcription primer (Supplementary Table S1) in a volume of 8 μl. Following this, 2 μl of 10 mM dNTPs were added and samples were incubated for 5 min at 37°C, before transferring them to ice for 2 min. A reverse transcription master mix was created by mixing 2 μl of 10× NTP minus buffer (0.5 M Tris–Cl pH 8.0, 750 mM KCl and 100 mM DTT), 4 μl of 5 M betaine, and 3 μl of 40 mM MnCl_2_ per sample. A total of 9 μl of the reverse transcription master mix was added to each sample. The samples were then incubated at room temperature for 2 min before the addition of 1 μl of 200 U/μl SuperScript II Reverse Transcriptase (Invitrogen). Reverse transcription was performed using the following program in a thermocycler: 10 min at 25°C, 90 min at 42°C, 10 cycles of 50°C for 2 min followed by 42°C for 2 min, and a final incubation of 70°C for 10 min. The resulting cDNA was purified using the Zymo DNA Clean and Concentrator 5 kit. A total of 30–50 ng of cDNA was combined with a final concentration of 0.2 mM dNTPs, 0.25 μM of gene segment 11-specific primer (Supplementary Table S1) and 20 U/μl of Q5 Hot Start High-Fidelity DNA Polymerase in Q5 DNA Polymerase buffer (NEB). Amplification was performed using the following program in a thermocycler: 98°C for 30 s, 20 cycles of 98°C for 10 s, 65°C for 30 s, and 72°C for 20 s, followed by a final incubation at 72°C for 2 min. DNA was purified by addition of a 1× volume of AMPure XP beads (Beckman-Coulter). Beads were separated on a magnet and washed twice with 80% ethanol before eluting in 10 μl of water. A total of 2 ng of DNA from the first amplification was combined with a final concentration of 0.2 mM dNTPs, 0.5 μM of a universal forward primer to add Illumina adaptor (Supplementary Table S1), 0.5 μM of a sample specific reverse primer to add a sample specific index and Illumina adaptor (Supplementary Table S1), and 20 U/μl of Q5 Hot Start High-Fidelity DNA Polymerase (NEB) in Q5 DNA Polymerase buffer (NEB). Amplification was performed using the following program in a thermocycler: 98°C for 5 min, 10 cycles of 98°C for 10 s, 66°C for 30 s and 72°C for 20 s, followed by a final incubation at 72°C for 2 min. DNA was purified by addition of a 0.8× volume of AMPure XP beads (Beckman-Coulter). Beads were separated on a magnet and washed twice with 80% ethanol before eluting in 10 μl of water. Libraries were then loaded on an Illumina MiSeq at 10 pM and run for 600 cycles (300 × 2).

### SHAPE-MaP data analysis

Sequencing reads produced from our mutational profiling experiments were analyzed using the ShapeMapper2 pipeline (26), version 2.1.4 using Bowtie2 version 2.3.4.3 as the aligner. ShapeMapper2 computes mutation rates for both treated and untreated samples by counting the mutations at each individual nucleotide along the transcript in comparison to the reference sequence (Supplementary data file 2). Reactivity is computed by subtracting the mutation rate of the untreated sample from the mutation rate of the treated sample. Statistical outliers are then excluded from further analysis and reactivities are normalized as previously described (27).

### RNA secondary structure modeling and forgi classification

All minimum free energy diagrams were generated using RNAStructure (28) by incorporating SHAPE reactivities as a pseudo free energy term with default parameters and using a maximum pairing distance of 150 nucleotides unless otherwise stated. Using connectivity tables (.CT) generated from RNAStructure we then used the Forgi python library to classify each nucleotide into a specific RNA secondary structure motif (29). These nucleotide classifications were generated from our SHAPE informed secondary structures derived from structure probing experiments of the RV transcriptome incubated together at an equimolar ratio.

### Shannon entropy modelling

Base-pairing probabilities for each transcript were predicted using RNAfold from the ViennaRNA package with the -p option (30). This option enables the calculation of the partition function and base pairing probability matrix (31). We predicted the base-pairing probability matrix for each RNA sequence R. Shannon entropy values (32), ${H_i}$ for all nucleotides $i \in R$ were then calculated using all base pair probabilities ${p_{ij}}$ of a paired nucleotide $i$ with $j$ across all potential paired nucleotides $R$ as follows:

$$\begin{equation*}{H_i} = - \sum\limits_{j = 1}^R {{p_{ij}}{{\log }_{10}}{p_{ij}}} \end{equation*}$$

A median of a 50-nucleotide sliding window was used to create the final Shannon entropy profiles for each transcript.

### RNA conservation analysis

All nucleotide sequences of rotavirus A (taxid: 28875) for NSP5, NSP4 and VP6 were extracted from the NCBI virus database (07.27.2021). Pairwise alignments of all sequences of each segment with the respective segment of the bovine RF strain used in our experiments (KF729678.1, KF729677.1, KF729658.1) were carried out using MAFFT with default parameters, and the Levenshtein distance for each pair was calculated (33,34). Sequences that had less than 90% sequence identity with our reference sequence, and/or that introduced gaps in the reference sequence with missing conserved terminal sequences were removed. This resulted in a total of 79 sequences for NSP5, 77 sequences for NSP4 and 328 sequences for VP6. The 90% sequence cut-off for NSP5, VP6 and NSP4 was chosen as appropriate following the recommendations for full-genome classification of rotaviruses by Matthijnssens *et al.* (35). Multiple sequence alignments for each segment were generated with MAFFT as described above, and these alignments were used to calculate the nucleotide conservation of the most abundant nucleotide at each position. All sequences, and the corresponding GenBank accession numbers are provided in the Supplementary data file 2. Since the nucleotides were generally conserved, we also computed the mean of a 10-nucleotide window to generate conservation heatmap plots to depict the variation in the nucleotide conservation between 0.75 and 1.00. RNA structures were created using RNAfold from the ViennaRNA package with the -p option to calculate the partition function and the –shape option to incorporate our SHAPE-MaP values in the prediction (30). These structures were then visualized and coloured using VARNA by reassigning the conservation values of each segment between 0 and 1 (36).

### Statistical analyses

Mutation rate distribution analysis and establishment of linear relationships between NSP2 titration points were analyzed using R studio (version 2021.09.1 + 372) and visualized with the ggplot2 package. Box plots and histograms comparing the log_2_(FoldChange) of RNA structural motifs was done using the python package MatPlotLib.

## RESULTS

### SHAPE-MaP derived structural analysis of rotavirus A transcriptome

**Figure 1.**
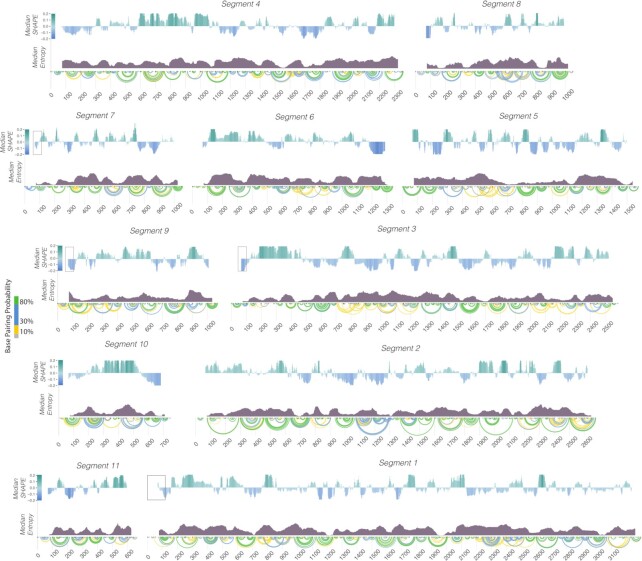
The Rotavirus transcriptome exhibits a diverse ensemble of RNA structures. (**A**) Median SHAPE reactivity of each of the 11 transcripts representing the rotavirus transcriptome averaged over two highly correlated (*R*^2^ ∼ 0.73–0.98) replicates. SHAPE-MaP experiments were carried out by incubating equimolar amounts of each RV segment transcript and probed with 5NIA, as described in Materials and Methods. A 50-nucleotide window was used to calculate median SHAPE values along each transcript and normalized to the overall median of the individual transcript. Areas of high median SHAPE (i.e. above zero, shown in green) represent conformationally flexible RNA regions. Areas of low median SHAPE (i.e. below zero, shown in blue) represent regions with high propensity to base-pair. Arc plots show pseudo-free energy derived base-pairing probabilities computed using nearest-neighbor RNAStructure parameters constrained by incorporating SHAPE reactivities (18,28). Probabilities of base-pairing are color-coded, with green arcs corresponding to the most probable base-pairs, and grey being the least probable ones. Boxed regions indicate conserved nucleotides. Conformational entropy plots (median Shannon entropy calculated with a 50-nucleotide sliding window) computed for RV transcripts are plotted underneath median SHAPE values. High Shannon entropy regions typically correspond to positions with alternative conformations, while low entropy regions identify sequences that are more prone to adopting a dominant MFE configuration.

Recently established reverse genetics approaches for rescuing viable rotaviruses utilize RVA transcripts produced by T7 polymerase-driven transcription (8). We utilized a set of plasmids designed for this virus rescue to produce the eleven RVA transcripts *in vitro* (23). The RNA produced was shown to be intact and homogenous (Supplementary Figure S1). An equimolar mix of the eleven RV transcripts (0.25 μM total RNA concentration) were incubated in RNA folding buffer (100 mM NaCl, 100 mM HEPES-Na, 0.5 mM MgCl2, pH 7) at 37°C for 30 min. Under these conditions, the eleven RVA transcripts did not form stable inter-molecular contacts in our previous single-molecule fluorescence studies (13). The RNA mix was then incubated with the SHAPE reagent 5-nitroisatoic anhydride (5NIA), as described in Materials and Methods (37). Conformationally flexible nucleotides exhibit high reactivities towards the electrophile 5NIA (37), thus allowing us to assess the RV transcriptome reactivity with single-nucleotide resolution. We performed at least two replicates of all SHAPE experiments, and SHAPE reactivities were reproducible with correlation coefficients for replicates in the range 0.73–0.98 (Supplementary Table S2). We quantified relative flexibility of each transcript using median SHAPE analysis (Figure 1A) as previously described (37–41). Regions of low median SHAPE are generally more likely to fold into single, well-defined conformations, while regions with above median SHAPE values generally adopt multiple conformations (29). We modeled the secondary structure and calculated base-pairing probabilities for the entire RV transcriptome (Figure 1) using our experimentally determined SHAPE reactivities averaged over both replicates as a pseudo-free energy term, as previously described (39,42,43).

**Figure 2.**
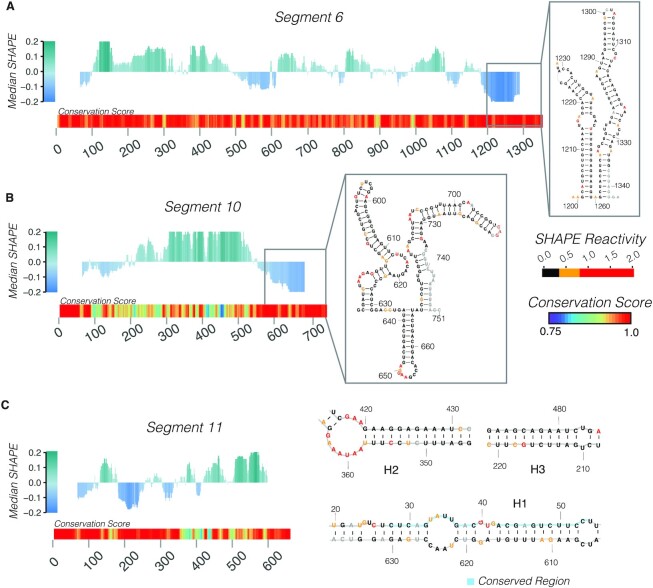
SHAPE-MaP derived models support covariation-based secondary structure models. (**A**) Median SHAPE reactivity of segment 6 in the presence of the RV transcriptome. Median SHAPE profile and base-pairing probabilities were calculated as described in Figure 1. Grey box indicates two terminal stem-loops that were predicted in segment 6 by Li *et al.* (5), and are further confirmed by our SHAPE-informed secondary structure model. Beneath the median SHAPE profile is a conservation score heatmap plot calculated using a 10-nt rolling window as described in Materials and Methods. (**B**) Median SHAPE-MaP profile and base-pairing probabilities of segment 10 in the presence of the RV transcriptome. Median SHAPE profile and base-pairing probabilities were calculated as described in Figure 1. Grey box highlights three stem-loops modelled in the 3′ terminal region of segment 10, as previously predicted (5). Additionally, these three loops are located in a highly-conserved region of segment 10 as indicated by the high conservation score. (**C**) SHAPE-MaP profile of segment 11 RNA shown along with the conservation heatmap plot (*left*), and the corresponding models of conserved RNA helices H2 and H3 (*right*) supported by the SHAPE-MaP data. Highly conserved nucleotides were previously predicted to participate in LRIs between terminal regions and our SHAPE-informed MFE model validates these predictions (5).

To validate our SHAPE-MaP probing data, we compared our SHAPE-informed secondary structure model of the segment 11 transcript to a predicted secondary structure model derived from our previously published RNA-RNA SELEX data (13). The analysis revealed that both models were broadly similar (PPV of 66.8% and sensitivity of 84.52%, Supplementary Figure S1). In addition, we compared our SHAPE-derived models with the secondary structure models generated using sequence conservation analysis alone. Our conservation analysis identified highly conserved regions present in segments 6, 10 and 11 (Figure 2). For group A rotavirus segments 6 and 10, the analysis of 328 unique VP6-coding and 77 NSP4-coding sequences, respectively (sequence selection criteria are described in Materials and Methods) has revealed multiple discrete regions of high conservation, most notably encompassing terminal nucleotides (Figure 2). In the segment 6 RNA, the conserved 3′ terminal sequences are predicted to fold into highly structured stem-loops (Figure 2A), fully supported by our SHAPE-MaP data. We further identified two previously predicted stem-loops located in the 3′ terminal region of segment 6 known to enhance translation (44). Similarly, segment 10 RNA was predicted to have a highly conserved terminal structure comprising stable stem–loops in the 3′ UTR (Figure 2B), fully in agreement with previous covariation analysis carried out by Li *et al.* (5). Moreover, analysis of the shortest segment 11 transcript reveals extended conserved sequences, broadly correlating with lower median SHAPE reactivity (Figure 2C), while our SHAPE-MaP data supports the presence of long-range interactions (LRIs, including conserved helices H1 to H3 shown in Figure 2C), in agreement with the prediction of Li *et al.* Finally, our probing data enabled us to identify the predominant LRI structure in segment 9 amongst the alternative models that had been previously proposed by Li *et al.* (Supplementary Figure S2). Collectively, these data reveal that all RV transcripts have structured 5′ terminal regions, expanding into coding regions, except for segments 2 and 5. Previously, it was found that the first 78 nucleotides in segment 1 and segment 7 are highly conserved (5). Additionally, the first 82 nucleotides and the first 94 nucleotides of segments 9 and 3, respectively, are conserved. All these regions of high conservation appear to be structured, i.e. with low median SHAPE reactivity, as seen in Figure 1A. In contrast, segments 2 and 5 both have unstructured 5′ terminal regions, with neither of these segments exhibiting sequence conservation in the 5′ terminal regions (5). Thus, our data support the notion that RNA structural pervasiveness is present within areas of high sequence conservation in RV genomes, and demonstrate that our experimental data are in good agreement with previously determined co-variation models.

**Figure 3.**
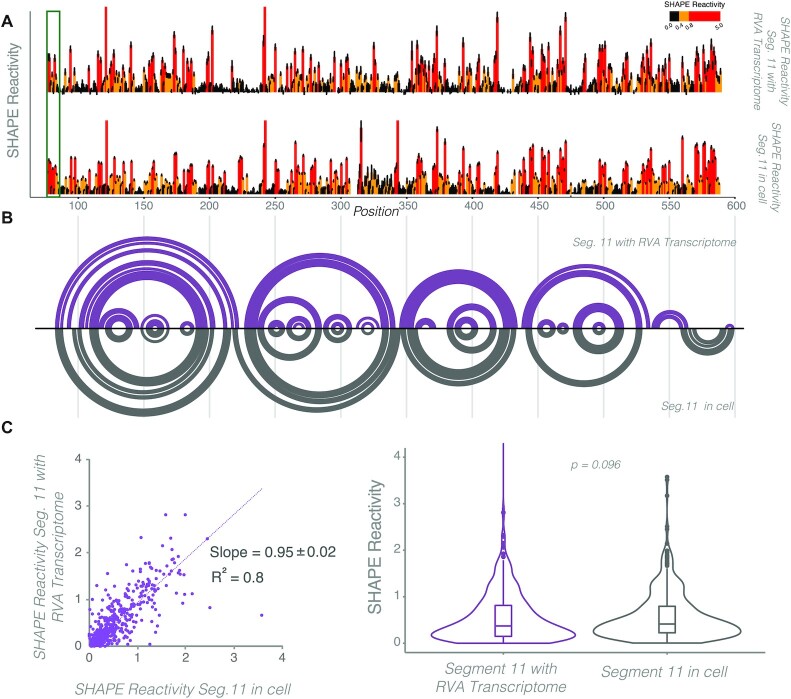
SHAPE-MaP analysis of *in vitro* transcribed segment 11 RNA *versus* in-cell transcript probing during RV infection. (**A**) Top panel: SHAPE reactivities of segment 11 transcript probed with 5NIA along with RV transcriptome. Bottom panel: in-cell SHAPE reactivity profile of segment 11 RNA probed with 5NIA at 6 hours post infection, as described in Materials and Methods. Individual bars represent SHAPE reactivities at single nucleotide resolution, black bars indicate nucleotides with the lowest reactivities (<0.4) and red bars denote nucleotides with the highest reactivities (>0.8). (**B**) Overlaid arc diagrams show similarities and differences between base-pairing in segment 11 RNA in the context of the complete RV transcriptome *in vitro* (Top) versus in the context of RV-infected cells (Bottom). Minimum free energy arc diagrams were generated by incorporating SHAPE-MaP data as a pseudo-free energy term in RNAStructure, as described in Figure 1. (**C**) Left: Scatterplot correlation analysis reveals that segment 11 transcript SHAPE reactivities between the *in cell* and *in vitro* transcribed RNA conditions are highly correlated (*R*^2^= 0.8). Right: Violin plots showing distribution of SHAPE reactivities of segment 11 RNA when probed *in cell* or *in vitro*. Boxes represent the 25th/75th interquartile range, and medians are shown as central bands. SHAPE reactivities are not significantly different as assessed by Kruskal-Wallis test (*P* < 0.05).

To validate our findings in cells, we also carried out SHAPE-MaP on RV-infected cells at 6 h post infection, i.e. prior to dsRNA synthesis when the majority of the viral RNA is single-stranded. Overall, SHAPE-MaP-guided structures of both segment 11 RNAs probed *in vitro* and *in vivo* were similar (Figure 3), revealing that the RV RNA conformational ensembles in cells are remarkably comparable to the ones obtained *in vitro* (*R*^2^ = 0.8). We also noted that the highly conserved sequence immediately before the start codon of NSP6 encoded by segment 11, had high SHAPE reactivity both *in vitro* and in cells (Figure 3A, green box), strongly supporting the notion that this region may be highly accessible for translational initiation, as proposed previously (5).

### Mutation rate analysis reveals that NSP2 increases segment 11 RNA backbone flexibility

**Figure 4.**
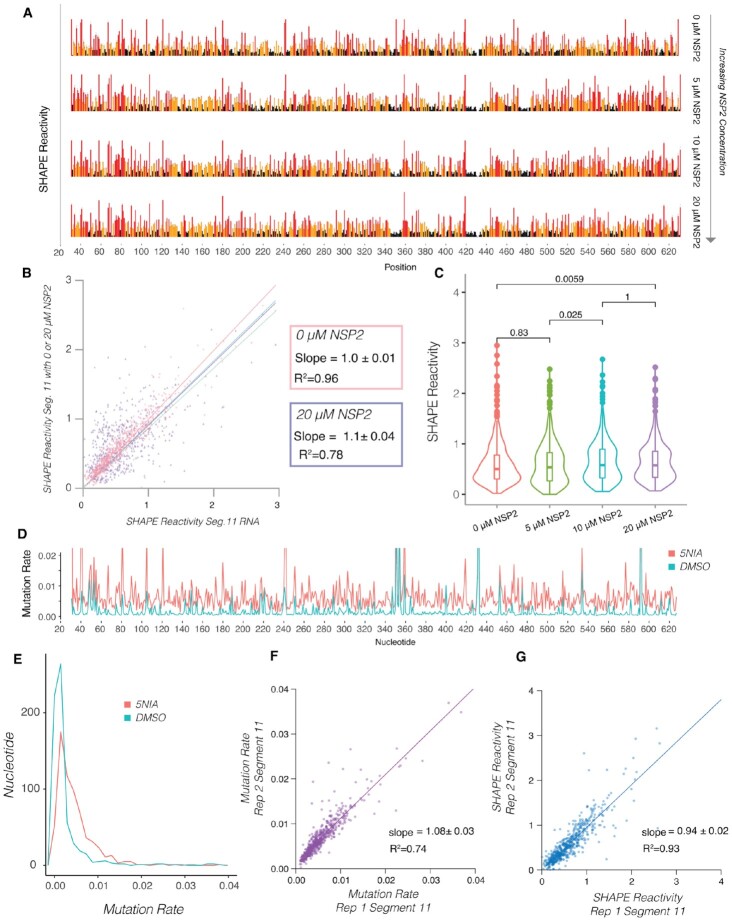
NSP2 addition does not result in detectable changes in the median SHAPE reactivities of segment 11 RNA. (**A**) Segment 11 was incubated with increasing amounts of NSP2 (0, 5, 10 and 20 μM) then probed with 5NIA, as described in the Methods section. SHAPE reactivities for each of the listed titration points show no obvious difference due to the normalization protocol. (**B**) Scatterplots showing the linear relationship between segment 11 SHAPE reactivity (x axis) and segment 11 SHAPE reactivity when incubated with increasing amounts of NSP2 (y-axis). Note high correlation between the data (*R*^2^ = 0.96, *R*^2^ = 0.78), and the linear relationship exhibits no significant change between the titration points. (**C**) Violin plots comparing distribution of SHAPE reactivities of segment 11 RNA upon incubation with NSP2. Boxes represent the 25th/75th interquartile range, and medians are shown as central bands. Addition of 20 μM NSP2 changes the distribution of SHAPE reactivities, while the median SHAPE reactivity does not significantly change, as assessed by the Kruskal-Wallis test (*P* < 0.05). Note there is no significant difference between the conditions despite a significant global increase in mutation rate. (**D**) Raw mutation rates of segment 11 RNA structure probing experiment. The red line is representative of segment 11 RNA mutation rates induced by the incubation with 12.5 mM of the modifying reagent, 5-nitroisatoic anhydride (5NIA), and the blue is the measured mutation rate across the segment 11 transcript treated with dimethyl sulfoxide (DMSO) as a control. Normalization of these mutation rates results in the SHAPE profiles shown in panel A. (**E**) Histogram representation of distribution of nucleotide mutation rates in treated and untreated samples of segment 11 RNA shown in panel D. (**F**) Scatterplot showing linear relationship of mutation rate data between reproducible replicate experiments. The slope when comparing both replicates is near 1, showing that mutation rate data are highly correlated (R^2^ = 0.74, slope = 1.08 ± 0.03). (**G**) Scatterplot showing linear relationship of SHAPE-MaP data between reproducible replicate experiments. SHAPE-MaP data are highly correlated (*R*^2^ = 0.93, slope = 0.94 ± 0.02).

To measure the effects of NSP2 on RVA transcripts, we began by incubating *in vitro* transcribed segment 11 RNA (0.67 kb) with increasing amounts of NSP2 and carried out SHAPE chemical probing (Figure 4A). Individual SHAPE reactivities of nucleotides across the segment 11 transcript are represented in a bar chart, where high SHAPE reactivities above 0.7, red bars, indicate a flexible nucleotide and nucleotides with low SHAPE reactivities below 0.5, black bars, represent constrained nucleotides. Visual inspection of these data does not reveal any obvious large differences in the SHAPE profiles. This is confirmed when plotting the SHAPE reactivities of segment 11 alone against when segment 11 was incubated with increasing amounts of NSP2) (Figure 4B). The slopes for all the titration points are close to 1, and the data remain highly correlated with *R*^2^ = 0.78 for the 20 μM NSP2 end point (Figure 4B, purple line), which is within expected replicate to replicate variability (Supplementary Table S3). Further analysis of SHAPE reactivities of segment 11 RNA in the presence of increasing concentrations of NSP2 reveals only a subtle change in the global median of SHAPE reactivities (Figure 4C). Thus, SHAPE-MaP analysis detects only minimal structural rearrangements without a significant apparent global change in SHAPE reactivity.

SHAPE reactivities are calculated from raw mutation rates (Figure 4D and E). The mutation rate of the untreated sample (Figure 4D, DMSO control) is subtracted from the mutation rate of the treated sample (Figure 4D, 5NIA) for each individual nucleotide. Statistical outliers are removed, and the remaining mutation rate intensities are normalized so all reactivities fall between 0 (highly constrained) to 2 (highly flexible) (26,27). Thus, if NSP2 binding uniformly increases reactivities of all bases, the apparent SHAPE reactivities would remain unchanged, while the mutation rates would uniformly increase for all bases. This prompted us to investigate the raw mutation rates. As expected, raw mutational rate analysis confirmed that nucleotides in the 5NIA-treated samples are more reactive compared to the DMSO-treated control. Furthermore, both mutation rate and SHAPE reactivity analyses replicates are highly correlated and have a slope near one (Figure 4F and G). We therefore hypothesized that if NSP2 has a global effect on backbone flexibility, it would effectively be normalized out and only an analysis of the raw mutation rates could reveal such effects.

**Figure 5.**
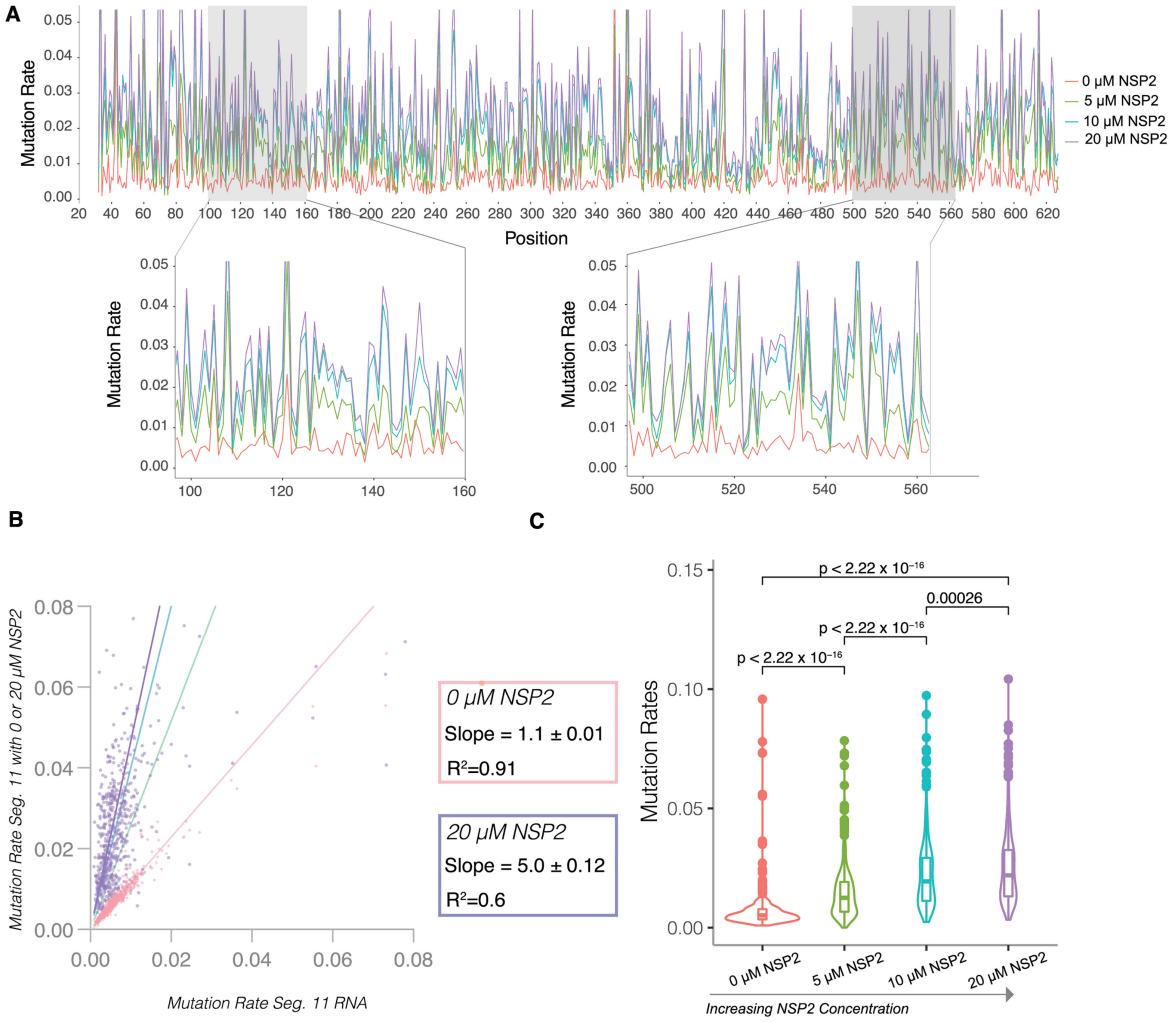
NSP2 uniformly increases segment 11 RNA mutation rate in a concentration- dependent manner. (**A**) Segment 11 was incubated with increasing amounts of NSP2 (0, 5, 10 and 20 μM), then probed with 5NIA, as described in Materials and Methods. Higher mutation rates reveal increased flexibility in the RNA backbone, while lower mutation rates indicate less conformationally flexible nucleotides. Note a uniform increase in mutation rates proportional to NSP2 concentration. Zoomed-in: representative regions of segment 11 RNA. (**B**) Violin plots comparing distribution of mutation rates of segment 11 RNA upon incubation with NSP2. Boxes represent the 25th/75th interquartile range, and medians are shown as central bands. Note, both change in mutation rate distribution and significant change in median mutation rate. Significance values were calculated using Kruskal–Wallis test (*P* < 0.05). (**C**) Mutation rates of segment 11 RNA (x-axis) plotted against mutation rates of segment 11 RNA in the presence of increasing amounts of NSP2 to reveal linear relationship with distinct slopes. The slope increase as NSP2 is titrated in, while the linear dependence persists, suggesting a global change in the RNA flexibility in response to saturation with NSP2.

Remarkably, the addition of NSP2 resulted in a concentration-dependent increase in the distribution of nucleotide mutation rates (Figure 5A), in agreement with our hypothesis. The pattern of the mutation rates for all bases however remained similar (Figure 5A), following a very similar trend observed in the normalized SHAPE reactivities (Figure 4A). When we plotted the mutation rate upon addition of NSP2, we observed a strong linear correlation for increasing concentrations of the RNA chaperone, with the slope increase up to 5.0$ \pm 0$.1 for the highest 20 μM NSP2 concentration tested (Figure 5B). We did not attempt higher NSP2 concentrations, as those have been previously shown to cause RNA aggregation rather than promote strand-annealing reactions (13). The mutation rate increase in the presence of NSP2 is further confirmed by our analysis of distribution of mutation rates in NSP2-concentration dependent manner (Figure 5C). Thus, NSP2 has a global effect on segment 11 backbone reactivity as measured by mutation rate, that is masked upon normalization when SHAPE reactivities are computed using the standard normalization procedure. Specifically, the quartile normalization step used when deriving SHAPE reactivities from mutational profiling data (27,38) has the unintended effect of masking these global changes in reactivity in the data.

Finally, to ensure that the changes in mutation rate are mediated by NSP2 and not perturbations caused by the addition of protein, we also incubated segment 11 RNA with 20 μM BSA and found no changes in mutation rate or SHAPE reactivity (Supplementary Figure S3), suggesting the observed effect is specific to NSP2.

### NSP2 uniformly increases backbone flexibility in RV transcripts

**Figure 6.**
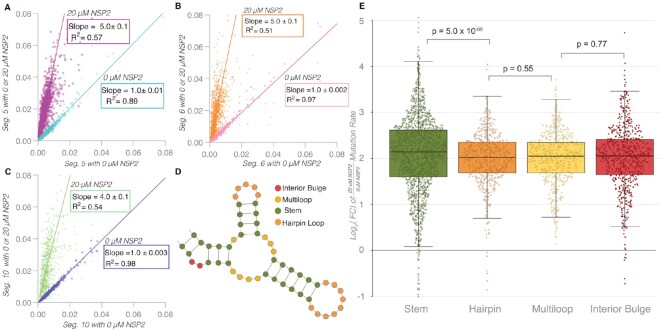
NSP2 uniformly increases backbone flexibility in RV RNAs. (**A**–**C**) Mutation rates of individual RNAs (segment 5, segment 6 and segment 10, respectively) alone (x axis) plotted against the mutation rates of the RNAs incubated with 20 μM NSP2 (y axis). (**D**) Schematic of structural motifs found within an RNA. Using SHAPE-MaP-informed secondary structure models generated as shown in Figure 1, individual nucleotides (color-coded) were categorized into each of the different structural modalities described: interior bulge (red), multiloop (yellow), stem (green), and hairpin loop (orange) using the Forgi library (Materials and Methods). (**E**) NSP2-mediated mutation rate change analysis for individual structural motifs as described in panel D. Log_2_(FC) denotes log_2_(mutation rates induced by 20 μM NSP2 divided by the mutation rates without NSP2). A vast majority of log_2_(FC) values are positive. Statistical significance was assessed using Kruskal-Wallis test. Stem-forming nucleotides involved in base-pairing have a higher average log_2_(FC) than those located within predicted forming hairpins, multiloops and interior bulges.

To assess if NSP2 increases backbone flexibility across different RV segments, or if the global increase in backbone flexibility is specific to segment 11 RNA, we examined *in vitro* transcribed segment 5, 6, and 10 transcripts with increasing concentrations of NSP2. We found a similar increase in mutation rate in segments 5, 6, and 10 as segment 11. As can be seen in Figure 6A–C, the mutation rate 20 μM endpoint of the titration yields slopes >4 for segments 5, 6, 10, respectively. Collectively, these data suggest NSP2 increases mutation rate across RV transcripts indiscriminately.

### NSP2 preferentially increases mutation rate in base-paired structural motifs

The overall pattern of mutation rates remains highly correlated upon addition of up to 20 μM NSP2 (Figure 6A–C) for each segment, as does the SHAPE data. Therefore, we applied mutation rate analysis to determine if NSP2 has any structural specificity at transcriptome scale, by categorizing all nucleotides using the Forgi library classifications (interior bulge, multiloop, stem, and hairpin loop) as described in the Methods (Figure 6D) (29). These classifications are based on the SHAPE-directed minimum free energy secondary structures reported in Figure 1.

To compare nucleotides in the four different structural contexts, we computed the log_2_(Fold Change) of the mutation rate at 20 and 0 μM NSP2 respectively. As can be seen in Figure 6E, a vast majority of nucleotides have log_2_(FC) >0, consistent with the positive slopes we observed in the scatter plots in Figure 6A–C. Remarkably, we also observed a statistically significant increase in the log_2_(FC) for paired nucleotides (stem) compared with unpaired (hairpin loop, multi-loop, and interior bulge, Figure 6E). The advantage of computing log_2_(FC) in this way is that it allows quantitative comparison of nucleotides with differing mutation rates. Thus, our data show that although the effect of NSP2 on mutation rates is uniformly positive, NSP2 has a larger relative effect on the flexibility of stems versus unpaired nucleotides, consistent with its helix-destabilizing activity.

### Structural consequences of rotaviral transcriptome on log_2_(FC)

**Figure 7.**
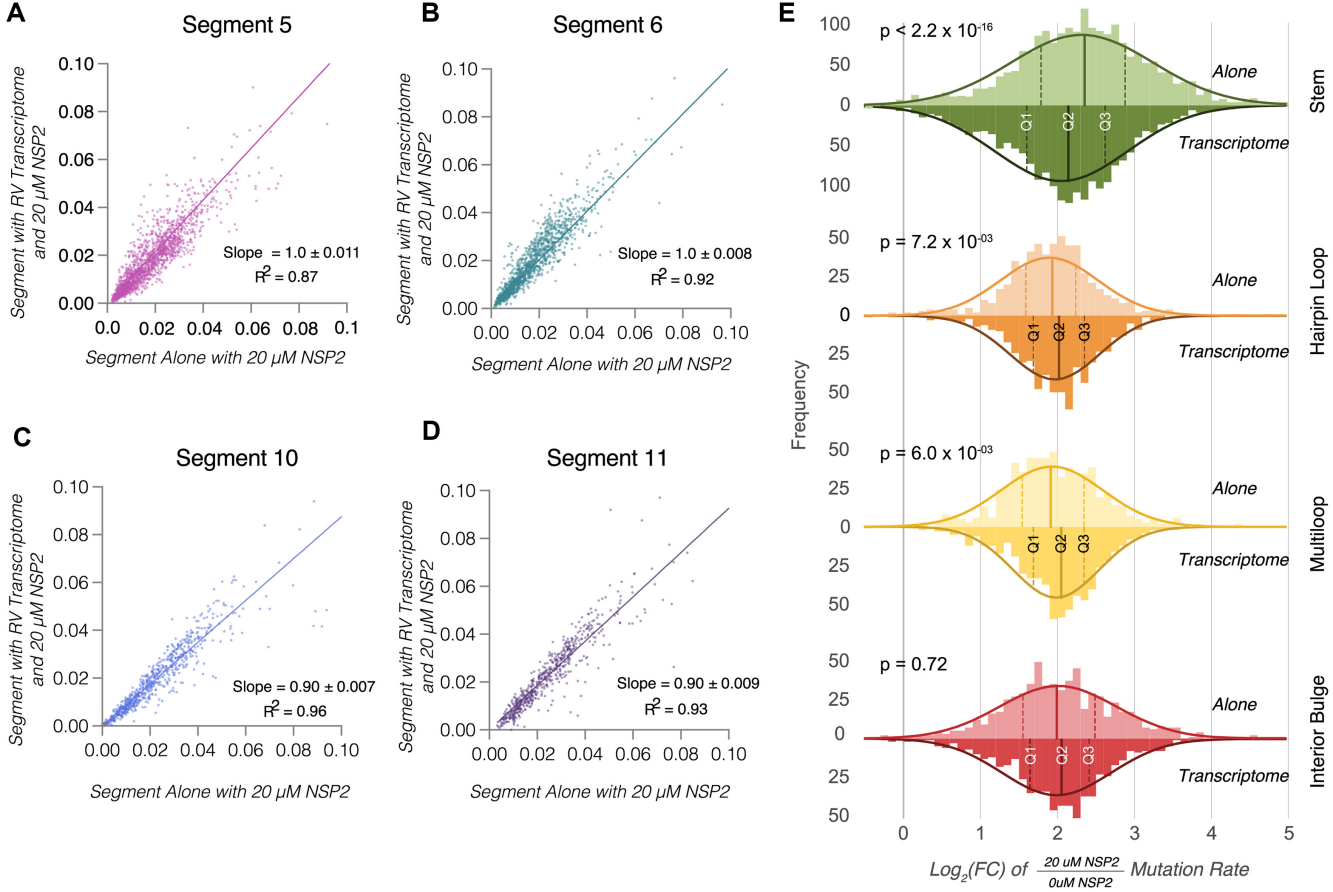
Changes in global backbone flexibility in RV transcriptome mediated by NSP2. Addition of NSP2 to an equimolar mix of 11 distinct RV transcripts results in global increase of mutation rates across all segments. (**A**–**D**) Mutation rates of individual RNAs incubated with an RNA mix representing the RV transcriptome in the presence of 20 μM NSP2(y-axis) plotted against mutation rates of the RNA alone in the presence of 20 μM of NSP2 (x-axis) to reveal highly correlated data. (**E**) Analysis of NSP2-mediated mutation rate changes in the presence or absence of the rotavirus transcriptome. Comparison of distribution of NSP2 mediated mutation rate changes (i.e. the log_2_ of the quotient of the mutation rates in the presence of 20 μM NSP2 over the mutation rates for RNA without NSP2) for each the structural motif (stem, hairpin loops, multiloops, interior bulge, as described in Figure 6D) for RNAs alone and RNAs in the context of the transcriptome. Q1 and Q3 represent the 25th and 75th interquartile range, respectively, and the median, Q2, is labeled shown as solid lines. Statistical significance was assessed using Kruskal-Wallis test.

As we have previously shown, at sub-nanomolar concentrations, rotaviral segments do not appear to interact intermolecularly when NSP2 is absent (13). Indeed, when we compare segments probed alone versus those probed in the presence of all 11 segments (Supplementary Figure S4) we observe highly correlated mutation rates and slopes close to 1. We were therefore interested to see if our log_2_(FC) analysis can detect any statistical differences at transcriptome scale when all eleven segments are incubated with NSP2. We therefore probed the entire transcriptome in the presence of increasing concentrations of NSP2. As can be seen in Figure 7A–D, the 20 μM NSP2 mutation rates are highly correlated in both the absence (x-axis, alone) and presence (y-axis, transcriptome) of the other segments, with slopes near 1 in all cases. We also computed slope and *R*^2^ for increasing concentrations of NSP2 and report these in Table 1, and all transcripts exhibit a similar concentration dependent trend. This suggests very little difference in the overall structure of these segments in the presence or absence of the transcriptome.

**Table 1.** NSP2 increases mutation rates indiscriminately across all RV RNAs

	5 μM NSP2/0 μM NSP2		10 μM NSP2/0μM NSP2		20 μM NSP2/0 μM NSP2	
Segment	Slope	*R* ^2^	Slope	*R* ^2^	Slope	*R* ^2^
*Segments probed in the context of RV transcriptome*						
Segment 1	2.1 ± 0.01	0.84	3.3 ± 0.03	0.67	5.4 ± 0.06	0.54
Segment 2	2.3 ± 0.02	0.81	3.3 ± 0.04	0.70	5.4 ± 0.07	0.55
Segment 3	2.3 ± 0.02	0.90	3.2 ± 0.03	0.79	4.5 ± 0.06	0.69
Segment 4	2.2 ± 0.02	0.92	3.3 ± 0.03	0.81	5.5 ± 0.07	0.63
Segment 5	2.4 ± 0.02	0.90	3.0 ± 0.03	0.88	4.2 ± 0.06	0.83
Segment 6	2.1 ± 0.02	0.84	2.9 ± 0.05	0.73	5.9 ± 0.1	0.44
Segment 7	2.0 ± 0.03	0.88	2.9 ± 0.05	0.79	5.7 ± 0.1	0.74
Segment 8	2.1 ± 0.03	0.97	2.9 ± 0.04	0.96	4.9 ± 0.1	0.86
Segment 9	2.1 ± 0.04	0.84	3.0 ± 0.04	0.79	4.2 ± 0.07	0.73
Segment 10	2.2 ± 0.05	0.80	2.8 ± 0.08	0.65	5.9 ± 0.2	0.59
Segment 11	1.9 ± 0.04	0.88	2.8 ± 0.08	0.73	6.6 ± 0.2	0.53
*Segments probed alone*						
Segment 5	2.9 ± 0.03	0.87	2.8 ± 0.03	0.88	4.8 ± 0.05	0.69
Segment 6	2.4 ± 0.04	0.72	3.7 ± 0.06	0.56	4.8 ± 0.1	0.51
Segment 10	2.1 ± 0.04	0.70	3.3 ± 0.08	0.42	3.9 ± 0.1	0.54
Segment 11	2.6 ± 0.07	0.84	4.0 ± 0.1	0.67	4.6 ± 0.1	0.65

Slopes indicate NSP2 induced increases in mutation rate for each segment calculated by dividing the mutation rate at each point in the titration experiment by the mutation rate of the segment without NSP2 as measured by 5NIA chemical probing. As NPS2 concentration increases, as does the slope. We calculated slope values for the entire RV transcriptome at increasing concentrations of NSP2 and for Segments 5, 6, 10, and 11 alone.

However, when we compared the log_2_(FC) distributions for segments 5, 6, 10 and 11 alone and in the presence of the rest of the viral transcriptome (Figure 7E), we observed a structure-specific difference. The analysis shows that the log_2_(FC) is systematically higher for paired nucleotides (as observed in Figure 7E) for both individual transcripts alone and in the presence of transcriptome. However, there are subtle but statistically significant differences in the quartile distributions for paired and unpaired nucleotides. Notably, the quartile distributions of mutation rates are significantly lower for paired nucleotides (green) in the presence of the transcriptome, while for hairpin and multi-loops they are significantly higher (orange, yellow and red). Similarly, for interior bulges, the increase is positive, but not significant, likely due to the small number of nucleotides in this conformation relative to loops and stems.

In the context of our previous results, this analysis reveals a subtle but important result that could be explained by transient intermolecular interactions between unpaired nucleotides in the context of the transcriptome. First, neither the addition of NSP2 nor the transcriptome has any detectable effect on the pattern of the SHAPE reactivities (Figure 3A). Thus, it can be concluded that NSP2 is not systematically refolding the RNA in a way that is detectable by chemical probing. Despite fairly subtle changes, our analysis suggests that NSP2 exerts a larger effect on the log_2_(FC) for paired nucleotides. Remarkably, in the presence of NSP2, paired bases are significantly more reactive towards 5NIA compared to unpaired nucleotides. However, their median reactivity decreases in the presence of the remaining rotaviral transcriptome (Figure 7E, green), consistent with the proposed model that the RNA chaperone NSP2 facilitates inter-molecular RNA-RNA interactions by exposing complementary sequences that are normally sequestered by the local structure (13).

Taken together, these data show that quantitative analysis of mutation rates can detect subtle but important structural preferences in promiscuous RNA-binding proteins like NSP2 at transcriptome scale, even if they do not appear to have strong structural or sequence preference (45) or when analyzing the normalized SHAPE-MaP data.

## DISCUSSION

The transcriptome-wide chemical structure probing of eleven rotavirus A transcripts data presented here validates previous computational and co-variance models of these RNAs, providing first experimental support to enable modelling of these structures. In addition, we have identified multiple well-defined structured regions of low SHAPE reactivity across the entire transcriptome (Figure 1). Our data suggest that RNA structure is pervasive in rotaviral segments and not limited to untranslated terminal regions. Pervasive RNA secondary structures are a feature of all RNA viruses studied to date using chemical probing including flaviviruses (46), influenza (41), HIV (9), alphaviruses (10,39), and coronaviruses (47,48) Interestingly, in-cell probing of one of the segments, gene segment 11 transcript, revealed a very similar chemical reactivity profile to that seen *in vitro*. Interestingly, this result contrasts with the observations that transcripts of influenza A viruses exhibit distinct reactivities when refolded *in vitro* vs mRNAs that appeared to be less structured in cells (49). It should be noted that the structures we have identified here only exist prior to genome packaging, i.e. before they are replicated into fully double-stranded RNA form inside the virion (1,50,51), thus precluding the analysis of the RNA structure *in virio*. Nonetheless, the analysis of rotavirus transcriptome has generated insights into the pervasiveness and dynamic nature of RNA structures for all RV transcripts that all contain regions of low SHAPE reactivity ranging from 50 to 200 nucleotides. Moreover, we used SHAPE-MaP to evaluate how NSP2, a key RNA chaperone involved in RNA assortment and packaging in rotaviruses, may interact with these pervasive structures.

Despite multiple biophysical and biochemical studies that have demonstrated helix-destabilizing activity of NSP2 with shorter RNA substrates (12,13,15), surprisingly, incubation of any of the rotaviral segments with even molar excesses of NSP2 had no significant effect on apparent SHAPE reactivity. At first, these results appeared to be at odds with our previous biophysical studies of the RNA chaperoning effect of NSP2 on small RNA stem-loops that revealed a melting of the structure (13,15). However, direct analysis of adduct-induced mutations via mutational profiling allowed us to observe the previously unknown global effect of NSP2 on the rotaviral transcripts.

Indeed, the quartile normalization used in the SHAPE-MaP data processing effectively masks any large global changes in mutation rates. Thus, although next generation sequencing enables unprecedented throughput, detailed understanding of the experimental system (i.e. specific vs non-specific RNA binding, RNA:protein binding stoichiometry, complementary biophysical data supporting RNA conformational changes) should always be considered for careful data interpretation. Furthermore, visualization of the raw mutational data during analysis should always be carried out to reveal any changes in the RNA backbone dynamics that might be normalized out in subsequent data processing steps. Additionally, we carried RNA probing using the established SHAPE reagent 1M7 in the presence and absence of NSP2. Our results with 1M7 reagent confirm the 5NIA data, suggesting that upon addition of NSP2, the RNA backbone of segment 11 transcripts increases its reactivity as quantified by the mutation rate (Supplementary Figure S5).

Having established that we observe a very significant, global, and NSP2 concentration-dependent increase in mutation rate upon addition of the RNA chaperone, we decided to evaluate whether we observe any structural specificity. Previously, it has been suggested that with small RNAs, NSP2 preferentially binds unpaired nucleotides with sub-nanomolar affinity (13). Our classification analysis of bases as paired or unpaired nucleotides allowed us to observe that the log_2_(Fold Change) in mutation rate upon addition of NSP2 was more positive for paired nucleotides across the entire RV transcriptome (Figure 6). Our previous studies suggest that NSP2 preferentially binds unpaired RNA regions (45), therefore the higher log_2_(Fold Change) for paired regions does not reflect any structural preference in binding of NSP2. Rather, our mutational profiling analysis indicates that NSP2 has a larger relative effect on paired nucleotide's flexibility, consistent with its role as an RNA chaperone that promotes RNA-RNA interactions by making sequestered complementary sequences more accessible for inter-molecular base-pairing. It is important to note that the increase in mutation rate indicates a global change in backbone flexibility, but does not change the overall SHAPE pattern. To directly interrogate any base pairing changes mediated by NSP2, we performed an analogous NSP2 titration using dimethyl sulfate (DMS), which methylates unpaired adenines and cytosines, thus directly reporting on the propensity for base-pairing. The NSP2-mediated increase in mutation rate is consistent between DMS- and 5-NIA-treated samples (Supplementary Figure S6). Indeed, when treated with DMS, we observed that stem-forming paired nucleotides experience a more significant increase in log_2_(FC) in the presence of NSP2 (Supplementary Figure S6), further confirming helix-destabilizing effect of NSP2 as the result of increased RNA backbone flexibility. It should be noted that further in-depth analyses followed by an extensive functional validation will be required to identify the nucleotides involved in the assembly of the eleven distinct RV transcripts. Recent studies of the assortment and packaging of the 8 RNA segments in influenza A viruses suggest that in IAVs such interactions are likely to be highly redundant, and potentially transient in nature (41,52), thus making their identification and validation a particularly challenging task. Nevertheless, understanding the mechanistic role of promiscuous RNA-binding proteins with RNA chaperone activity will help our endeavors to elucidate the RNA assembly pathways leading to a stoichiometric segment packaging in these viruses.

The data presented here further support a model in which NSP2 is able to capture folded RNA stem–loop structures within a single RNA-binding groove, resulting in general relaxation of RNA structure without fraying of the RNA stem termini (15). This allows the RNA structure to be sufficiently flexible that it can adopt a conformation compatible with stabilization of sequence-specific, potentially transient RNA-RNA contacts. By binding to multiple RNAs concurrently via surface-exposed positively charged grooves, NSP2 octamers act as matchmakers of complementary sequences, promoting intermolecular RNA–RNA interactions (15). While NSP2 promiscuously binds any ssRNA with sub-nanomolar affinity, the proposed mechanism would require NSP2-RNA interactions to be transient to allow sampling of multiple interacting RNA partners. We propose that on a transcriptome level, NSP2 achieves its RNA chaperone activity by globally increasing RNA backbone flexibility, while its RNA-RNA matchmaking is governed by specific, yet to be revealed, complementary sequences.

## DATA AVAILABILITY

Raw sequencing reads are available on the NCBI SRA database under BioProject ID PRJNA791398. All SHAPE data used in this study are available in Supplementary data file 1 in SNRNASM format.

## Supplementary Material

gkac738_Supplemental_Files
