# Supplementary material for: Rotavirus RNA chaperone mediates global transcriptome-wide increase in RNA backbone flexibility
Source: Nucleic Acids Res. 2022 Sep 5;50(17):10078–92. doi: 10.1093/nar/gkac738 (PMC9508848; doi:10.1093/nar/gkac738)
Supplement: gkac738_Supplemental_Files [file gkac738_supplemental_files.zip › Supplemental_data_05.04.22-compressed.pdf]

Supporting data for manuscript

‘Rotavirus RNA chaperone mediates global transcriptome-wide increase in RNA backbone flexibility’.

Aaztli Coria, Anastacia Wienecke, Michael L Knight, Daniel Desirò, Alain Laederach, Alexander Borodavka

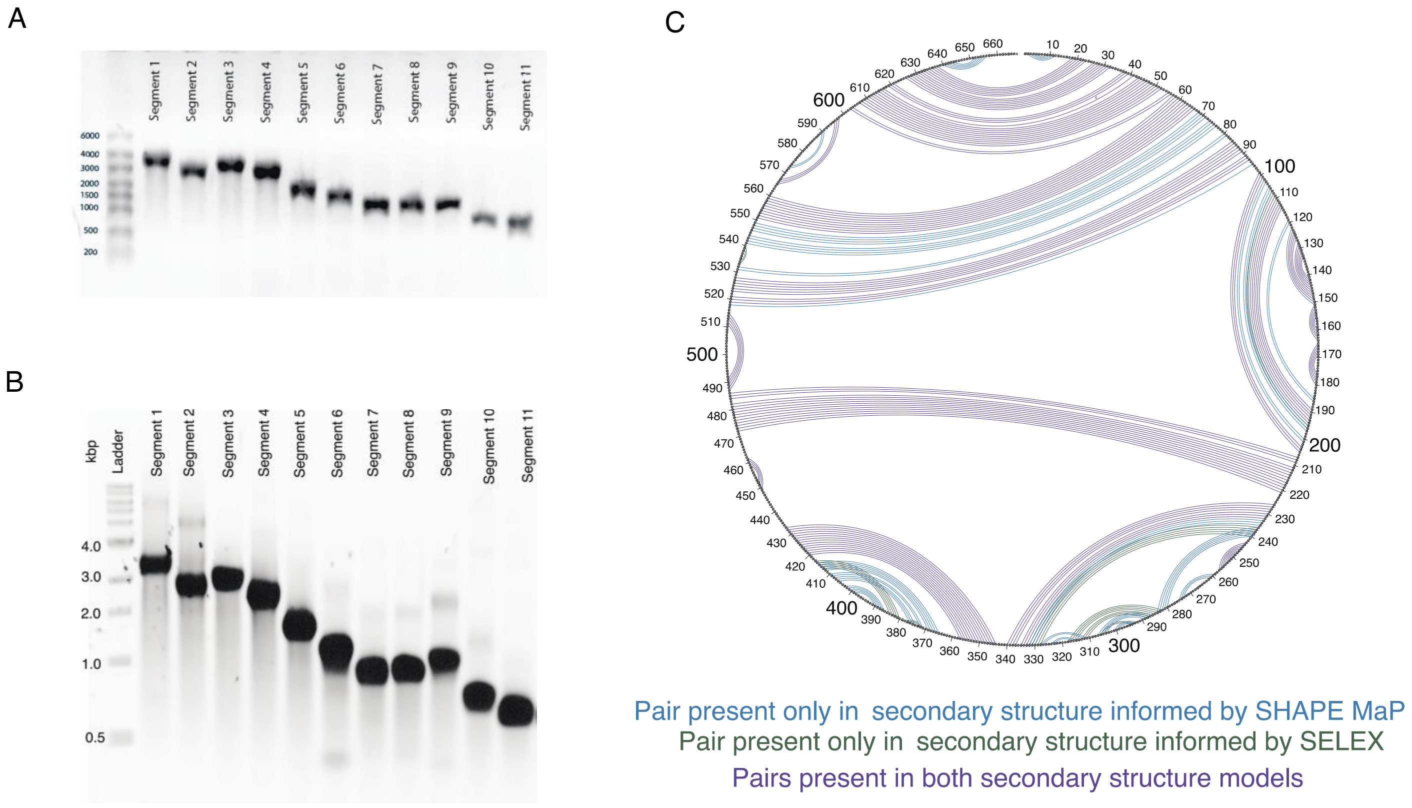

Supplementary Figure 1. Rotavirus RNA templates used in this study and comparison of previously predicted segment 11 secondary structure with our experimentally validated segment 11 secondary structure. (A) Denaturing (7.2% v/v formaldehyde-MOPS buffer) 1% agarose gel of *in vitro* synthesized RV transcripts for RV gene segments 1 – 11 (Bovine RVA strain RF, as described in Materials and Methods). 1 µg of each transcript were resolved on a gel. These RNAs were then aliquoted, stored in -80, and used for the studies described in this paper. (B) Native agarose gel (1% agarose, TBE buffer) of *in vitro* synthesized RV transcripts, 1 µg of each RNA were prepared the same way as for SHAPE-MaP analysis (see Materials and Methods), and resolved on a 1% agarose gel. (C) Circle plot comparison of our SHAPE-informed secondary structure diagram of Segment 11 and a SELEX informed segment 11 secondary structure prediction we previously proposed (Borodavka et al. 2017; Reuter and Mathews 2010). Individual lines in the circle represents a predicted base pair, base pairs in purple represent base pairs that both secondary structure models share, blue lines are base pairs only present in the SHAPE-MaP derived secondary structure we present here, and green lines represent base pairs in the SELEX informed secondary structure model. We found that both models were broadly similar with a PPV of 66.8% and sensitivity of 84.52%.

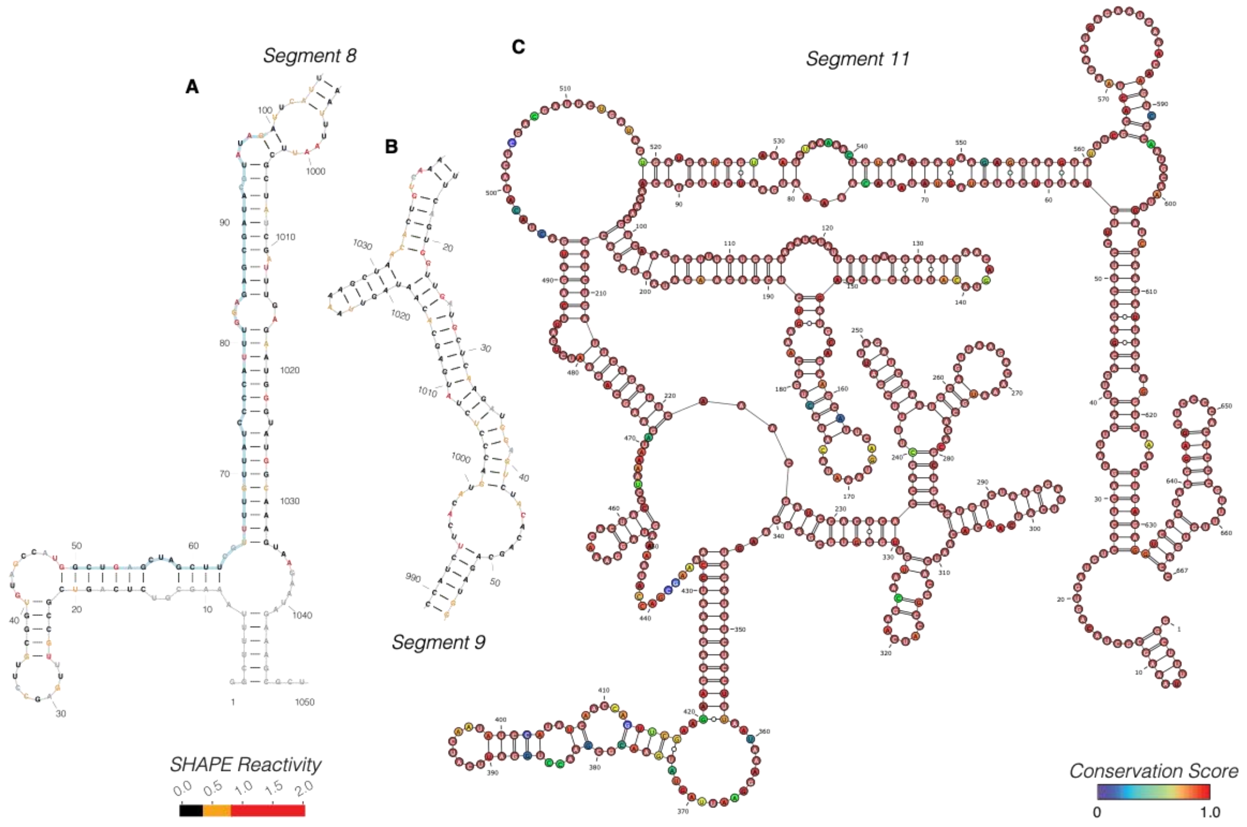

Supplementary Figure 2. SHAPE-MaP derived models are in agreement with covariation-based models of RV transcripts' secondary structures.

(A) Segment 8 (Bovine RV strain RF) MFE model computed using SHAPE reactivities constrains. The stem shown here includes highly conserved nucleotides within segment 8 (nts 50-98), in agreement with previously proposed covariation models (Li et al. 2010). (B) Segment 9 transcript MFE model using SHAPE reactivities as a pseudo free energy term, this model supports a predominant LRI structure amongst two alternative models that had been previously suggested but not experimentally verified in the study by Li *et al*. Individual nucleotide SHAPE reactivities are colored according to the heatmap shown below (high reactivity values are shown in red). (C) SHAPE-constrained MFE model of segment 11 transcripts with nucleotides colored by conservation. Red nucleotides are the most conserved nucleotides while blue nucleotides are less conserved. Conservation analysis was carried out using 79 unique full-length group A RV NSP5 sequences as described in Materials and Methods.

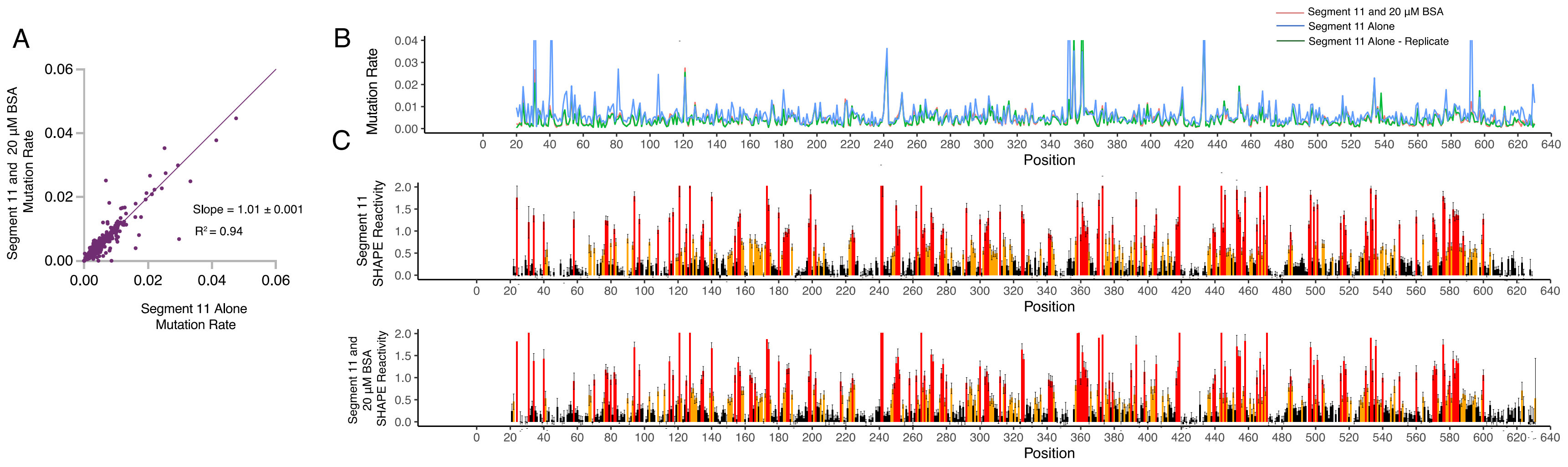

Supplementary Figure 3. BSA has no impact on segment 11 transcript secondary structure and does not alter 5NIA-induced mutation rates. (A) Scatter plot comparing mutation rate of Segment 11 transcript alone and Segment 11 transcript in the presence of 20 μM BSA. The slopes between the two data sets are almost identical (slope 1.0 ± 0.001), with the correlation of $R^2 = 0.94$. (B) Nucleotide mutation rates used for generating the scatter plots in (A) plotted across the sequence of segment 11 transcript. Red line represents the mutation rate of Segment 11 with 5NIA and 20 μM BSA, the blue line represents segment 11 alone, and the green line represents a replicate of Segment 11 alone. (C) Normalized SHAPE reactivities of the mutation rates shown in panel B. Top: SHAPE reactivity profile of Segment 11 alone. Bottom: SHAPE reactivity profile of Segment 11 incubated with 20 μM BSA.

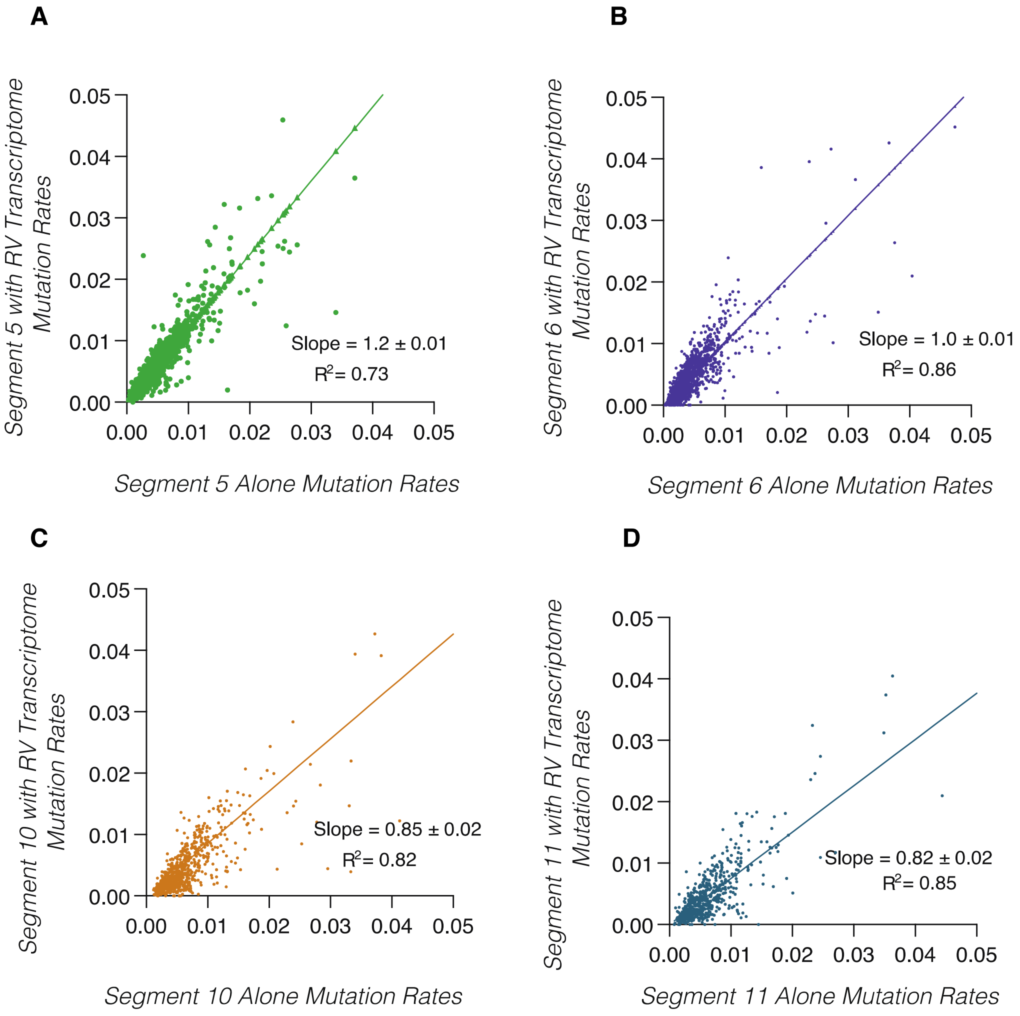

Supplementary Figure 4. Incubation with additional RV transcripts does not change RNA mutation rates. (A-D) Scatter plot comparing the mutation rate data of segments 5, 6, 10, 11 alone (x axis) with the mutation rates of the same segments in the context of the entire transcriptome (y axis). The data sets are highly similar with slopes around 1 and high correlations R^2 close to 1.

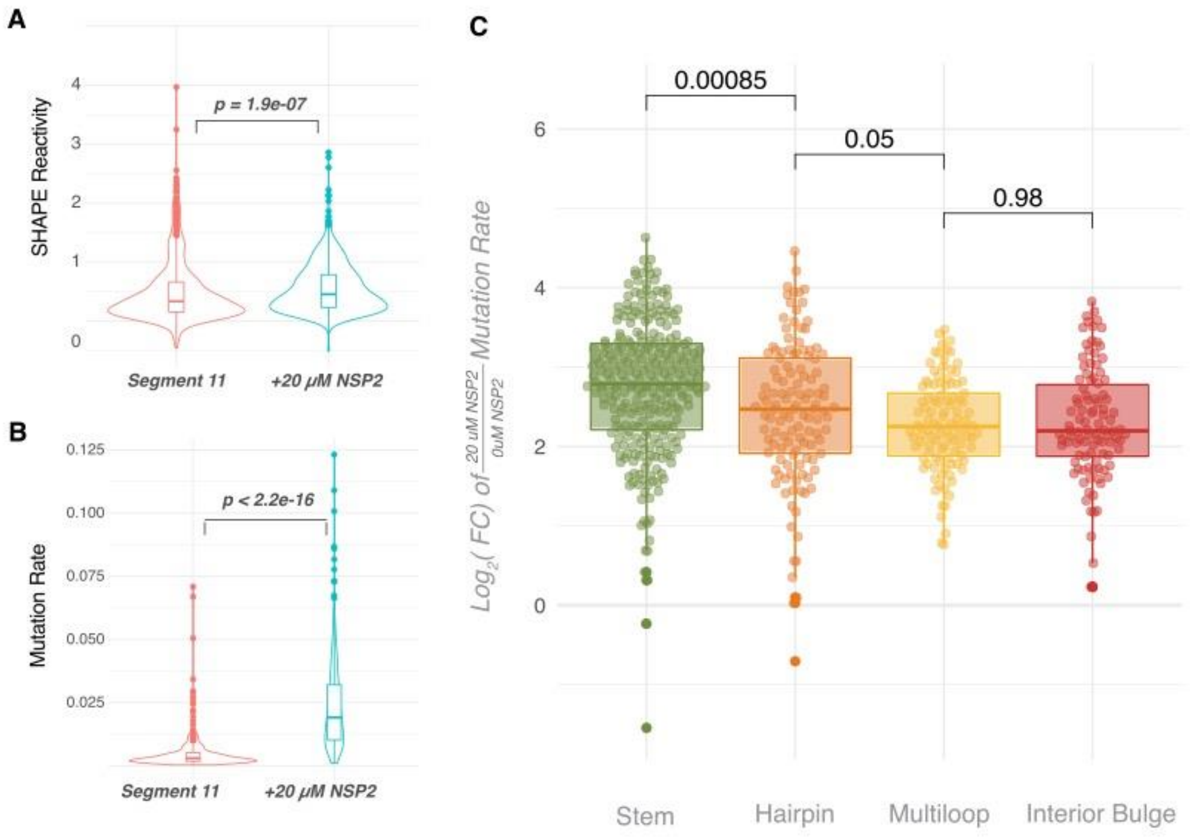

Supplementary Figure 5. NSP2 increases both 1M7-induced and 5-NIA-induced mutation rates in protein concentration-dependent manner. (A-B) Violin plots comparing the distribution of 1M7-induced mutation rates (10 mM 1M7, as described in Materials and Methods) of segment 11 transcript when incubated alone *vs* RNA with 20 µM NSP2. Boxes represent the 25th/75th interquartile range, and medians are shown as central bands. Significance values were calculated using Kruskal-Wallis test ($p < 0.05$). (C) NSP2-mediated mutation rate change analysis for individual structural motifs as previously described using $\log_2$(FC) analysis applied to our 1M7 probing data. The majority of $\log_2$(FC) values are positive, indicating that NSP2 increases RNA mutation rate as measured by 1M7 following a similar trend to 5NIA. Significance values were calculated using Kruskal-Wallis test showing nucleotides found within stems have a higher average $\log_2$(FC) than those located within single-stranded motifs similar to our findings using SHAPE reagents.

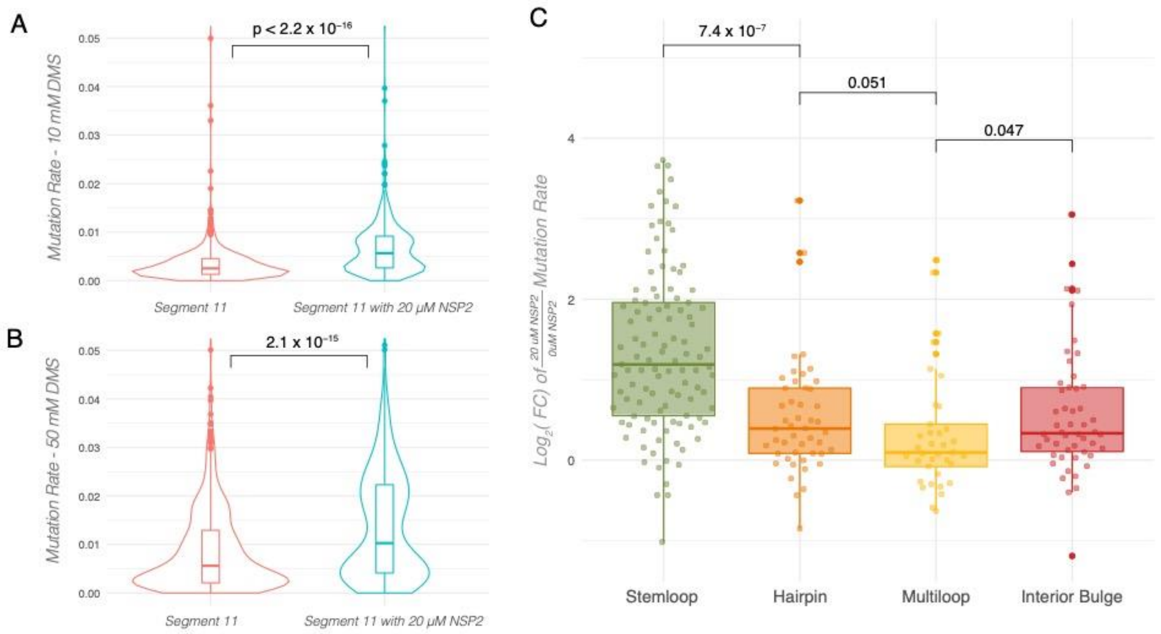

Supplementary Figure 6. NSP2 increases segment 11 DMS mutation rate. (A-B) Violin plots comparing distribution of DMS induced mutation rates of segment 11 when incubated alone and with 20 μM NSP2 when probed with either 10 mM or 50 mM DMS. DMS methylates the Watson-Crick base-pairing edge of unpaired adenine and cytosine bases. Boxes represent the 25th/75th interquartile range, and medians are shown as central bands. Significance values were calculated using Kruskal-Wallis test ($p < 0.05$) (C) NSP2-mediated mutation rate change analysis for individual structural motifs as previously described using $\log_2$(FC) analysis applied to our DMS probing data. The majority of $\log_2$(FC) values are positive, indicating that NSP2 increases RNA mutation rate as measured by DMS. Significance values were calculated using Kruskal-Wallis test, showing nucleotides found within stems have a higher average $\log_2$(FC) than those located within single-stranded motifs similar to our findings using SHAPE reagents.

Name	Sequence
RT_S11	CACTGTAGCGCTTTAAAGCC
1st_Amp_Fwd	CCCTACACGACGCTCTTCCGATCTNNNNNGCGCTACAGTGATGTCTCTCAG
1st_Amp_Rev	GACTGGAGTTCAGACGTGTGCTCTTCCGATCTNNNNNCGATCAATTGCATTGCGACTTG
2nd_Amp_Universal	AATGATACGGCGACCACCGAGATCTACACTCTTTCCCTACACGACGCTCTTCCG
2nd_Amp_Primer_#1	CAAGCAGAAGACGGCATACGAGATCGTGATGTGACTGGAGTTCAGAC
2nd_Amp_Primer_#2	CAAGCAGAAGACGGCATACGAGATACATCGGTGACTGGAGTTCAGAC
2nd_Amp_Primer_#3	CAAGCAGAAGACGGCATACGAGATGCCTAAGTGACTGGAGTTCAGAC
2nd_Amp_Primer_#4	CAAGCAGAAGACGGCATACGAGATTGGTCAGTGACTGGAGTTCAGAC
2nd_Amp_Primer_#5	CAAGCAGAAGACGGCATACGAGATCACTGTGTGACTGGAGTTCAGAC
2nd_Amp_Primer_#6	CAAGCAGAAGACGGCATACGAGATATTGGCGTGACTGGAGTTCAGAC
2nd_Amp_Primer_#7	CAAGCAGAAGACGGCATACGAGATGATCTGGTGACTGGAGTTCAGAC
2nd_Amp_Primer_#8	CAAGCAGAAGACGGCATACGAGATTCAAGTGTGACTGGAGTTCAGAC
2nd_Amp_Primer_#9	CAAGCAGAAGACGGCATACGAGATCTGATCGTGACTGGAGTTCAGAC
2nd_Amp_Primer_#10	CAAGCAGAAGACGGCATACGAGATAAGCTAGTGACTGGAGTTCAGAC
2nd_Amp_Primer_#11	CAAGCAGAAGACGGCATACGAGATGTAGCCGTGACTGGAGTTCAGAC
2nd_Amp_Primer_#12	CAAGCAGAAGACGGCATACGAGATTACAAGGTGACTGGAGTTCAGAC
2nd_Amp_Primer_#13	CAAGCAGAAGACGGCATACGAGATTGTTGACTGTGACTGGAGTTCAGAC
2nd_Amp_Primer_#14	CAAGCAGAAGACGGCATACGAGATACGGAACTGTGACTGGAGTTCAGAC
2nd_Amp_Primer_#15	CAAGCAGAAGACGGCATACGAGATTCTGACATGTGACTGGAGTTCAGAC
2nd_Amp_Primer_#16	CAAGCAGAAGACGGCATACGAGATCGGGACGGGTGACTGGAGTTCAGAC
2nd_Amp_Primer_#18	CAAGCAGAAGACGGCATACGAGATGTGCGGACGTGACTGGAGTTCAGAC
2nd_Amp_Primer_#19	CAAGCAGAAGACGGCATACGAGATCGTTTCACGTGACTGGAGTTCAGAC
2nd_Amp_Primer_#20	CAAGCAGAAGACGGCATACGAGATAAGGCCACGTGACTGGAGTTCAGAC
2nd_Amp_Primer_#21	CAAGCAGAAGACGGCATACGAGATTCCGAAACGTGACTGGAGTTCAGAC

Supplementary Table 1 List of primers and primer sequences used in this study.

	Rep 1 vs Rep 2: 0 µM NSP2		Rep 1 vs Rep 2: 5 µM NSP2		Rep 1 vs Rep 2: 10 µM NSP2		Rep 1 vs Rep 2: 20 µM NSP2	
Segment	Slope	R^2	Slope	R^2	Slope	R^2	Slope	R^2
Segments probed in the context of RV Transcriptome								
Segment 1	0.96 ± 0.01	0.91	0.92 ± 0.01	0.91	0.97 ± 0.003	0.97	1.0 ± 0.003	0.96
Segment 2	0.94 ± 0.01	0.91	0.93 ± 0.01	0.93	0.96 ± 0.004	0.95	1.0 ± 0.004	0.96
Segment 3	0.90 ± 0.01	0.87	0.87 ± 0.004	0.93	1.0 ± 0.004	0.97	1.0 ± 0.004	0.97
Segment 4	0.96 ± 0.01	0.91	0.92 ± 0.02	0.91	0.98 ± 0.004	0.97	1.0 ± 0.004	0.97
Segment 5	0.92 ± 0.01	0.92	0.94 ± 0.01	0.90	1.0 ± 0.004	0.97	0.96 ± 0.004	0.97
Segment 6	0.90 ± 0.01	0.78	0.85 ± 0.01	0.82	0.97 ± 0.01	0.95	0.92 ± 0.004	0.97
Segment 7	0.94 ± 0.01	0.88	0.88 ± 0.01	0.88	1.0 ± 0.01	0.97	1.0 ± 0.004	0.98
Segment 8	0.90 ± 0.01	0.80	0.89 ± 0.01	0.85	0.98 ± 0.01	0.95	0.96 ± 0.01	0.97
Segment 9	0.90 ± 0.01	0.88	0.89 ± 0.01	0.90	0.98 ± 0.01	0.96	0.96 ± 0.01	0.97
Segment 10	0.86 ± 0.02	0.73	0.80 ± 0.02	0.77	0.97 ± 0.01	0.92	0.92 ± 0.01	0.97
Segment 11	0.93 ± 0.02	0.80	0.86 ± 0.02	0.83	0.97 ± 0.01	0.92	0.90 ± 0.01	0.97
Segments probed alone								
Segment 5	0.89 ± 0.01	0.79	1.0 ± 0.02	0.68	0.93 ± 0.01	0.78	0.95 ± 0.01	0.93
Segment 6	0.84 ± 0.02	0.64	1.0 ± 0.01	0.94	1.0 ± 0.01	0.95	0.98 ± 0.01	0.92
Segment 10	1.0 ± 0.01	0.97	1.0 ± 0.004	0.99	1.0 ± 0.004	0.99	1.0 ± 0.003	0.99
Segment 11	0.94 ± 0.02	0.93	0.97 ± 0.01	0.96	0.99 ± 0.003	0.99	0.96 ± 0.01	0.99

Supplementary Table 2 Similarity and correlation between SHAPE-MaP reactivities of data sets of 11 segments all together and of segments 5, 6, 10, and 11 alone.

	Rep 1 vs Rep 2: 0 µM NSP2		Rep 1 vs Rep 2: 5 µM NSP2		Rep 1 vs Rep 2: 10 µM NSP2		Rep 1 vs Rep 2: 20 µM NSP2	
Segment	Slope	R^2	Slope	R^2	Slope	R^2	Slope	R^2
Segments probed in the context of RV Transcriptome								
Segment 1	0.97 ± 0.003	0.97	1.1 ± 0.003	0.97	0.95 ± 0.003	0.97	0.80 ± 0.003	0.97
Segment 2	0.98 ± 0.003	0.97	1.0 ± 0.003	0.97	0.95 ± 0.004	0.96	0.80 ± 0.003	0.97
Segment 3	0.97 ± 0.004	0.96	1.0 ± 0.004	0.97	0.95 ± 0.003	0.98	0.93 ± 0.003	0.98
Segment 4	1.0 ± 0.01	0.79	1.0 ± 0.01	0.93	0.98 ± 0.01	0.90	0.92 ± 0.01	0.94
Segment 5	1.0 ± 0.01	0.92	1.0 ± 0.004	0.97	0.96 ± 0.003	0.98	0.96 ± 0.004	0.98
Segment 6	0.99+0.01	0.95	1.1 ± 0.01	0.96	1.0 ± 0.01	0.85	0.98 ± 0.01	0.96
Segment 7	0.94+0.01	0.97	1.1 ± 0.01	0.97	0.93 ± 0.01	0.98	0.96 ± 0.003	0.99
Segment 8	0.95+0.01	0.94	1.0 ± 0.01	0.96	0.95 ± 0.01	0.96	0.95 ± 0.01	0.98
Segment 9	0.95+0.01	0.97	1.1 ± 0.01	0.98	0.95 ± 0.004	0.98	0.95 ± 0.004	0.98
Segment 10	0.98 ± 0.01	0.93	0.90 ± 0.01	0.96	0.95 ± 0.01	0.93	0.98 ± 0.01	0.93
Segment 11	1.1 ± 0.06	0.98	0.96 ± 0.07	0.97	0.89 ± 0.01	0.94	1.1 ± 0.01	0.96
Segments probed alone								
Segment 5	1.3 ± 0.01	0.89	0.93 ± 0.01	0.84	1.0 ± 0.02	0.80	1.0 ± 0.01	0.94
Segment 6	0.98 ± 0.02	0.68	1.0 ± 0.01	0.90	1.2 ± 0.01	0.93	1.1 ± 0.01	0.91
Segment 10	1.0 ± 0.01	0.98	0.90 ± 0.004	0.99	0.94 ± 0.004	0.99	0.94 ± 0.003	0.99
Segment 11	1.1 ± 0.03	0.74	1.1 ± 0.02	0.89	0.98 ± 0.01	0.98	0.99 ± 0.01	0.98

Supplementary Table 3 Similarity and correlation between mutation rates of data sets of 11 segments all together and of segments 5, 6, 10, and 11 alone.
